# Editorial: Deciphering the microbiome's role in the progression of interstitial lung diseases

**DOI:** 10.3389/fmed.2026.1879251

**Published:** 2026-06-01

**Authors:** Roberto G. Carbone, Anne Marie Russell

**Affiliations:** 1 DIMI, University of Genoa, Genoa, Italy; 2 College of Medicine and Health, University of Birmingham, Birmingham, United Kingdom; 3 Interstitial Lung Disease (ILD) Centre, University Hospitals Birmingham NHS Trust, Birmingham, United Kingdom

**Keywords:** connective tissue disease (CTD), gut lung axis, interstitial lung disease, microbiome, sarcoidosis

Recent research highlights a significant connection between the gut-lung microbiome imbalance (dysbiosis) and interstitial lung disease (ILD). Changes in the intestinal microbiome may influence the gut–lung axis, leading to inflammation and immune dysregulation that contribute to alveolar interstitial injury and fibrosis.

The aim of this Research Topic is to report the relationship between gut dysbiosis and ILD through pathogenesis, metagenomics, genetics, proteomics, diagnosis and treatment. This includes key ILD subsets, specifically idiopathic pulmonary fibrosis (IPF), sarcoidosis, and connective tissue associated ILD (CTD-ILD) in relation to dysbiosis. Contributing authors also explore modern and traditional Chinese medicine approaches, review contemporary clinical research, propose future trials and importantly, describe the possible damage to gut-lung axis by fungal and bacterial infections.

IPF is associated with altered lung microbiome and reduced diversity, often exhibiting higher bacterial burden, which correlates with disease progression and mortality. The abundance of bacteria in broncho-alveolar lavage fluid has been associated with faster disease progression, adverse outcome, elevated pro-inflammatory cytokines, a decline in pulmonary function tests and decreased survival rate (1). Proteobacteria such as *Streptococcus, Prevotella*, and *Veillonella*, mostly found in the digestive tract, suggest that dysbiosis could drive chronic alveolar injury and inflammation in the fibrotic lung (1). Changes in lung microbiome are associated with acute exacerbations (AE-IPF) and associated increased morbidity and mortality. The presence of common microbes such as firmicutes (*Streptococcus, Veillonella*), proteo-bacteria, bacteroidetes (*Prevotella*), and actinobacteria in the IPF lung, contributing to pathogenesis and disease progression, provides a target for future diagnostic and therapeutic strategies (2).

Metagenomics contributes to the identification of antibiotic resistance genes providing important information on resistance mechanisms and plays an indispensable role in treatment approaches by improving antibiotic stewardship (Xu et al.). Metagenomics offer the potential to improve diagnostic accuracy in immuno-compromised or critically ill patients with complex infections optimizing antibiotic treatment selection and improving outcome (Xu et al.). Ma et al. (3), integrate metagenomics with the microbiome, demonstrating a pro inflammatory: anti-inflammatory imbalance in the respiratory microbiota during AE-ILD, enabling identification of infection-related exacerbations and clinical management.

The relationship between fungal agents and sarcoidosis is complex and not fully understood. Generalova et al. suggest that fungoma dysbiosis in blood and the respiratory tract serves as an antigenic disease biomarker offering new routes for diagnosis and treatment. Further, fungal diversity related to environmental and endogenous origins has been detected in blood and lung tissue samples. Prominent genera such as *Candida, Saccharomyces*, and *Penicillum* may contribute to infection etiology, immune-pathogenesis, or progression of sarcoidosis. Future studies should focus on functional activity, host interactions, and temporal dynamics of fungi to clarify their role, persistence and resolution in sarcoidosis. Anti-fungal treatment may be indicated in some cases (Generalova et al.) (4). Carbone et al. report on the relationship between gut microbiota and sarcoidosis. The Authors describe the history of lung microbiome including a special section dedicated to this emerging topic reporting data from 2010 to present and exploring the role of microbiome, its interaction with immune system associated with lung disease and related pathologies such as cancer and COVID-19 infection. The study shows that dysbiosis is an emerging and contradictory area of investigation where cause/effect inference is yet unclear. The disease is influenced by genetic factors which interact with the microbiome. Sarcoidosis is a complex disorder where genetics is combined with environmental triggers, microbial antigens and gut dysbiosis developing chronic, dysregulated immune response and granuloma production (5). Finally, proteomic research demonstrates a significant role in dysbiosis and protein expression contributing to development sarcoidosis and progression (5).

Current knowledge on dysbiosis in CTD-ILD is well-established and gut inflammatory and immunomodulatory molecules participate in lung disease development. Puppo et al. report microbiome alterations related to several CTD-ILD including: (i) Rheumatoid arthritis, (ii) Systemic sclerosis, (iii) Sjögren's Syndrome, (iv) Systemic lupus erythematosus, (v) Dermatomyositis. CTD-ILD studies were mainly focused on systemic sclerosis and indicate that gut-lung dysbiosis is closely related to the disease, increasing gut permeability, producing oxidative stress and leading to lung inflammation (Carbone et al.). Furthermore, a therapeutic approach with pro-biotics and high fiber diets showed a protective role in CTD-ILD by gut lung axis modulation.

Traditional Chinese medicine suggests Gand-du-quing (GDQ) decoction capable to ameliorate chronic airway inflammation regulating lung microbiome and reducing pathological changes and inflammatory exudation in pulmonary tissue. Further, GDQ treatment improves lung function as shown by respiratory parameters in GDQ treated group compared to atmospheric particulate matter (PM) exposed control group. Exploring some immune cells markers. Wu et al. found that imbalance of these cells is crucial in PM-induced lung injury. The Authors evaluated the effect of PM exposure on lung microbiome using gene sequencing and studied the effect of GDQ on lung microbiota. Results demonstrated that the diversity and richness of lung microbiota were higher in PM exposed group than in the control group. GDQ could reduce the increase in abundance and diversity of lung microbiota caused by PM exposure.

Microbiome profiling and treatment that selectively eliminates pathogenic bacterial genera while preserving beneficial commensals offers hope for timely diagnosis and personalized therapy and better outcomes in ILD.
